# The Influence of Feed-Supplementation with Probiotic Strain *Lactobacillus reuteri* CCM 8617 and Alginite on Intestinal Microenvironment of SPF Mice Infected with *Salmonella* Typhimurium CCM 7205

**DOI:** 10.1007/s12602-018-9413-z

**Published:** 2018-04-07

**Authors:** Soňa Gancarčíková, Radomíra Nemcová, Miroslav Popper, Gabriela Hrčková, Ľuboslava Sciranková, Marián Maďar, Dagmar Mudroňová, Štefan Vilček, Rudolf Žitňan

**Affiliations:** 1 University of Veterinary Medicine and Pharmacy, Košice, Slovakia; 2 Institute of Parasitology, Slovak Academy of Sciences, Košice, Slovakia; 3 National Agriculture and Food Centre - Research Institute of Animal Production, Nitra, Slovakia

**Keywords:** Alginite, Probiotic, *Lactobacillus*, *Salmonella*, Mice

## Abstract

Alginite is a non-ore raw material arising by fossilization of accumulated organic (algae) and inorganic material, particularly clay, carbonates, quartz, and amorphous modification of silicic acid in the aqueous environment. Humic acids as a component of organic portion of alginite are known for very good buffering ability which allows them to stabilise pH throughout the digestion system of animals, stimulate receptors of the immune system in intestinal villi against pathogenic bacteria, and support proliferation and activity of beneficial bacteria (lactobacilli, bifidobacteria, and similar). Our investigations focused on the influence of a probiotic strain in combination with alginite on intestinal microenvironment of SPF mice infected with *Salmonella* Typhimurium. The 66 female mice (BALB/c) used in our study were divided to four experimental groups, control NC1, control NC2 (alginite), IC (alginite + *Salmonella* Typhimurium CCM 7205_NAL_), LAB (*Lact. reuteri* CCM 8617 + alginite + *Salm.* Typhimurium CCM 7205_NAL_). The group supplemented with *Lact.reuteri* CCM 8617 and alginite showed significant reduction in growth of *Salm.* Typhimurium in mice faeces at 24 and 72 h (*P* < 0.001) post infection. The supplementation of additives affected positively also nitrogen, enzymatic, hepatic and energy metabolism of mice. The demonstrable positive influence of additives alleviated the negative impact of *Salm.* Typhimurium infection on the morphology investigated in the jejunum and ileum of LAB group of mice. The livers of mice treated with both alginite and *Lact.reuteri* CCM 8617 showed marked reduction of overall inflammation, hepatocyte necrosis and size of typhoid nodules.

## Introduction

Permanent influence of various risk factors on the organism and their concurrent action supporting development of diseases encouraged search for effective and easily available means capable of protecting the health of animals and people. One of the ways that can positively affect intestinal microbiocenosis and thus increase resistance of the macro-organism is the stimulation of the beneficial autochtonous intestinal microflora of the gastrointestinal tract (GIT) by administration of preparations of biotechnological and natural character. Probiotics as natural bioregulators help to maintain the balance of the GIT ecosystem by several mechanisms and prevent colonisation of the digestive tract by pathogenic bacteria [1]. The beneficial micro-organisms (probiotics) can be administered in the form of natural substances that occur in the natural environment and are often consumed and even deliberately searched for by wild animals as a food ingredient. Clay, peat and other substances of mineral or fossil origin may be a favourable feed supplement. Fossil minerals, such as smectite, bentonite, lignite, leonardite and alginite represent special substances able to detoxify the microenvironment but, primarily, some of them contain large amounts of humates which are important to both flora and fauna. Alginite is a non-ore raw material arising by fossilisation of accumulated organic (algae) and inorganic material, especially clay (montmorillonite, illite, smectite), carbonates (dolomite, calcite, aragonite), quartz, and amorphous modification of silicic acid in the aquatic environment. The organogenic sediment associated with oil shales arose by the action of a group of primitive yellow-green algae (*Botryococcus braunii*). It has a high natural humidity, plasticity, relatively low weight and high porosity. We presumed that these properties could be used for the cultivation of beneficial micro-organisms as alginite and its humic acids could form the basic skeleton for “solid state” fermentation. Humic acids, as a part of the organic material alginite also have a very good buffering capacity [2], thereby can stabilize the pH throughout the digestive system of animals, stimulate receptors of the intestinal immune system against pathogens [3] and support proliferation and activity of beneficial microflora (lactobacilli, bifidobacteria, etc.). They facilitate better utilization of nutrients from the diet by stabilization of gut microbiota [4]. According to Huck et al. [3], humates added to animal diet stimulate microbial growth and the resulting counts of micro-organisms may be relatively high in dependence on their species, cultivation medium and environmental conditions. Due to their macrocolloid structure, humic acids protect the stomach and intestinal mucosa, peripheral capillaries and damaged mucous cells. This results in reduced or completely impossible resorption of toxic metabolites in the case of presence of residues of harmful substances in feed [5, 6]. Humic acids prevent an excessive loss of water from the gut, important in the treatment of diarrhoea, dyspepsia and acute intoxications [4]. A significant positive property of humic acids is their low resorption in the gastrointestinal tract (up to 0.1%) and only slight toxicity for the organism after their partial resorption. The bulk of humic acids is eliminated via faeces from the organism, and a part of them may be degraded by the intestinal microflora [7]. The important fact is that they are natural products and environmentally friendly [8] what allows their risk-free use in animal production and human medicine.

The aim of our study was to investigate the influence of feed-supplementation of a probiotic strain *Lactobacillus reuteri* CCM 8617 and a fossile additive (alginite) on the intestinal environment, the physiological parameters and the liver histopathology of model SPF mice of BALB/c line, infected with *Salmonella* Typhimurium CCM 7205_NAL_.

## Materials and Methods

### Animals, Housing and Diet

The experiment was carried out on 66 specific pathogen-free (SPF) BALB/c female mice, (4 weeks old), obtained from Velaz s.r.o. (Prague, Czech Republic). The conventional SPF mice were transported by air in special transport containers to the experimental facilities of the Laboratory of gnotobiology, University of Veterinary Medicine and Pharmacy (UVMP) in Košice. After a thorough surface disinfection of the containers with peracetic acid, these were transferred to gnotobiotic isolators (Velaz s.r.o., Prague, Czech Republic). After subsequent venting of peracetic acid vapours, the mice were transferred to 12 breeding polypropylene cages, 6–7 mice/cage of the following dimensions: length 365 mm; width 207 mm; height 140 mm. The mice were divided to 4 experimental groups, control NC1 (*n* = 16), control NC2 (alginite, *n* = 16), IC (alginite + *Salmonella* Typhimurium CCM 7205_NAL_, *n* = 17), LAB (*L. reuteri* CCM 8617 + alginite + *Salmonella* Typhimurium CCM 7205_NAL_, n = 17). All experimental procedures were approved by the Ethics Commission of the University of Veterinary Medicine and Pharmacy (Košice, Slovakia). The animals were fed ad libitum complex mixed feed for mice in a barrier breeding system Altromin 1311 (Velaz s.r.o., Prague, Czech Republic). The diet contained (g/100 g diet) crude protein 22.5, crude fat 5.0, crude fibre 4.5, ash 6.5, calcium 0.9, magnesium 0.2, sodium 0.2, potassium 0.9 and phosphorus 0.7 (vitamin A 1500 IU, vitamin B1 1.8 mg, vitamin B2 1.2 mg, vitamin B6 0.9 mg, vitamin E 7.5 mg, vitamin D3 60 IU, vitamin C 3.6 mg) and had unlimited access to water kept in glass bottles. They were kept at temperatures maintained between 20 and 24 °C, with relative humidity of 45–65%, under a 12-h light/dark regimen. Lignocel 3-4S (Velaz s.r.o., Prague, Czech Republic) bedding intended for barrier breeding was used. The experiment lasted 14 days and was composed of a baseline (day 0), supplementation (days 1–14) and post-infection periods (days 8–14). The animals were assigned to the following four groups: control group NC1 (*n* = 16); alginite control group NC2 (*n* = 16) – group continuously supplemented with alginite in feed (10%); IC group (*n* = 17) – mice infected with a single dose [0.1 mL of 10^8^ CFU/mL in BHI broth (Brain Heart Infusion, Merck KGaA, Darmstadt, Germany)/mouse] of *Salmonella* Typhimurium CCM 7205_NAL_, administered per os individually on day 7 from the beginning of supplementation of feed with alginite (10%); LAB group (*n* = 17) – mice infected with *Salm.* Typhimurium CCM 7205_NAL_ on day 7 of study and supplemented with *Lactobacillus reuteri* CCM 8617 (1.75 ± 4.5 × 10^8^ CFU/g) and alginite in feed (0.05% and 10%, resp.) continuously for 14 days. Mice were monitored for changes in body weight, clinical condition, vital parameters and appetite throughout the study.

### Preparation of *Lactobacillus reuteri* CCM 8617 for Administration to Mice

The probiotic strain *Lactobacillus reuteri* L2/6 Biocenol™ was isolated from pig faeces at the Institute of microbiology and gnotobiology of the UVMP in Košice. The strain was identified at the Institute of physiology of farm animals of the Slovak Academy of Sciences (ÚF HZ, SAV) in Košice by means of a MALDI BioTyper™ system (Bruker Daltonics Inc., Massachusetts, USA) with score 2.044. The strain was deposited with Culture Collection (Masaryk University, Brno, Czech Republic) and assigned number CCM 8617. The strain produces exopolysaccharides (EPS+) and is resistant to gastric juice [9]. Spontaneous rifampicin-resistant isolates were obtained by inoculation of the night culture of the respective strain to MRS agar (BD MS) containing 30 μg/ml rifampicin (Sigma Chemical Co., Great Britain). The obtained resistant colonies were incubated anaerobically on plates (BBL GasPak™ Plus, BD, USA) at 37 °C for 3 days. By this procedure, a resistant form of probiotic strain *Lactobacillus reuteri* CCM 8617_RIF2_ was obtained. A lyophilised form of this strain prepared by means of Freeze Dryer CoolSafe (LaboGene, Lynge, Denmark) was added to mice feed (0.05%).

### Infectious Strain

The mice in groups IC and LAB were infected with *Salmonella* Typhimurium, Culture Collection strain CCM 7205 (Masaryk University, Brno, Czech Republic). Spontaneously nalidixin-resistant isolates of this strain were obtained by inoculation of the night culture of the respective strain to BGA agar (Brilliant Green Agar with Sulfadiazine, Laboratorios Conda, Spain) containing 25 μg/ml nalidixin (Sigma Chemical Co., Poole, Great Britain). The obtained resistant colonies were incubated aerobically at 37 °C for 24 h. By this procedure, we obtained a resistant form of *Salmonella* Typhimurium CCM 7205_NAL_.

### Fossile Additive

Pre-dried, ground alginite (Algivo, s.r.o., Lučenec, Slovakia) of grain size 1–1.3 mm, subjected to gamma-irradiation (Bioster, Veverská Bitýška, Czech Republic), was used as a fossile additive.

### Sampling Procedures

The experiment lasted 14 days. Fresh faecal samples were collected on days 1, 7, 10 and 14 of the study. At the end of the study, the mice were anaesthetized with sodium pentobarbital at a dose of 86 mg per kg body of weight and euthanized by cervical dislocation. Blood samples for haematological and biochemical analysis were obtained by retroorbital blood collection from anaesthetized animals. Contents of *caecum* were collected on day 7 after infection with *Salmonella* Typhimurium (day 14 of study). Samples of the digestive tract for microbiological examination, weight of internal organs (heart, liver, spleen, kidneys and lungs), and samples of *lobus caudatus hepatis* were obtained during post mortem examination on day 14 of the study from all groups.

### Microbiological Analysis

The samples of faeces, caecum, liver and spleen (1 g) were mixed with a sterile Ringer buffer (Merck, pH 7.0) and homogenised (3 min) using a Stomacher Lab Blender 80 (Seward Medical Limited, London, UK). Microbial populations were determined according to the standard microbiological methods by serial dilution on the following selective media: MRS agar (Merck, Darmstadt, Germany) for lactic acid bacteria; TSA agar containing 5% ram’s blood (BBL, Microbiology systems, Cockeysville, USA) and BGA with nalidixin in concentration of 25 μg/ml (Brilliant Green Agar with Sulfadiazine, Laboratorios Conda, Spain) for *Salmonella* Typhimurium CCM 7205_NAL_. Aerobic bacteria were incubated under aerobic conditions. Anaerobic bacteria were incubated in an anaerostat (BBL GasPak™ Plus, Becton, Dickinson and Company [BD], Maryland, USA) at 37 °C for 2 days. The viable counts were expressed as the log 10 of colony forming units (CFU) per millilitre (mL) of sample. The results are given as arithmetical means ± standard deviation (SD).

### Biochemical and Haematological Analysis

Blood plasma was collected to tubes containing menadione ethylenediaminetetraacetic acid (K3EDTA). Haematological analysis was carried out using a BC-2008 VET automatic analyser (Mindray, Shenzhen, China). An automated biochemical analyser Ellipse (AMS, Rome, Italy) and standard kits (Dialab, Prague, Czech Republic) were used to determine concentrations of the selected biochemical parameters: triacylglycerides; cholesterol; HDL-cholesterol; LDL-cholesterol; total protein; urea; albumin; activities of enzymes: aspartate aminotransferase (AST); alanine aminotransferase (ALT); alkaline phosphatase (ALP).

### Short Chain Fatty Acids (SCFA) Analysis

Faeces and caecum contents (0.5 g) were diluted in 25 ml deionised water, homogenised (stomacher; IUL Instruments) and filtered through a filter paper. An aliquot of 30 μl was used for analysis of SCFA (lactic, succinic, acetic, propionic, butyric, valeric acids) by capillary isotachophoresis (Isotachophoretic analyser ZKI 01, Radioecological Institute, Košice, Slovakia). A leading electrolyte of the following composition was used in the pre-separatory capillary: 10 mmol/l HCl + 22 mmol/l ε-aminocaproic acid+0.1% methylhydroxyethylcellulosic acid, pH = 4.3. A solution of 5 mmol/l caproic acid+20 mol/l histidine was used as a finishing electrolyte. This electrolytic system worked at 250 μA in the pre-separatory and at 50 μA in the analytic capillary.

### Determination of Morphometric Parameters by Light Microscopy

Samples of mucosa (1 cm^2^) were taken from the medial part of both the jejunum and ileum. The samples were fixed in 4% formalin solution. After rinsing with water, the samples were dehydrated in a graded series of ethanol (30%, 50%, 70%, 90%, 100%), cleared with benzene, saturated with and embedded in paraffin. Sections of 7 μm thickness (10 slices of each sample) were stained with haematoxylin/eosin and examined under a light microscope. The length of 30 villi and depth of 30 crypts was determined by a computer-operated Image C picture analysis system (Imtronic GmbH, Berlin, Germany) and the IMES analysis software, using a colour video camera (Sony 3, CCD, Tokyo, Japan) and a light microscope (Axiolab, Carl Zeiss Jena, Germany).

### Histology of the Liver

Samples of *lobus caudatus hepatis* were fixed in 4% paraformaldehyde in PBS (Amresco LLC, Solon, USA) for 72 h and embedded in paraffin. Finally, counter-staining of the liver tissue was performed with Harrison’s haematoxylin and eosin and the tissue was mounted in Histochoice mounting medium (Amresco LLC, Solon, USA). Morphometric analysis of the areas of inflammatory nodules were performed at × 200 magnification on a field screen (corresponding to the area of 0.146 μm^2^) using an Olympus Microscope BX51 and a Digital Analysis Imaging System Analysis Docu (Soft Imaging Systems 3.0, Prague, Czech Republic). After analysis of at least 30 screen fields of sections of the livers of mice from groups IC and LAB, the mean area of nodules per field (0.146 μm^2^) for each liver was calculated. Finally, the mean area of inflammatory nodules on the sections from all examined livers (*n* = 4 for both groups) was calculated and expressed as mean ± SEM.

### Statistical Analysis

Statistical analysis was performed using Statistic software GraphPad Prism 3.0 for Windows (GraphPad Software, San Diego, USA). The most results are expressed as means ± SD. The data were evaluated statistically by one-way analysis of variance (ANOVA), followed by a multiple comparison Tukey’s test. Significant differences between the groups of mice were tested using analysis of variance and unpaired Student’s *t* test. The significance level was set to (*P* < 0.05).

## Results

### Total Body Weight and Relative Weight of Internal Organs

On day 14 of the experiment, the highest mean total body weight of BALB/c mice was recorded in control groups NC1 (18.64 ± 0.26 g) and NC2 (18.38 ± 0.27 g) (Table 1) while the mice included in the infected groups (IC, LAB) showed lower mean total body weight. The mean total weight of animals from group IC (infected animals supplemented with fossile additive) differed significantly from that of mice from both control groups NC1 (*P* < 0.01) and NC2 (*P* < 0.05). While the mean total weight of mice from IC group recorded on day 14 of study was the lowest, the relative weight of examined organs (liver, spleen, kidneys and lungs) was the highest in this group compared to non-infected control animals (NC1, NC2) (Table 1). The most pronounced difference in the relative weight of organs in both infected groups (IC, LAB) was observed in the weight of the liver and spleen which significantly differed (*P* < 0.001) in comparison with both control groups (NC1, NC2). In comparison with control group not supplemented with additives (NC1), the mice from IC group showed significantly higher (*P* < 0.05; *P* < 0.01; *P* < 0.001) mean relative weights of parenchymatous organs (left kidney, right kidney and lungs, respectively). Similar significantly higher relative weight of organs was recorded in this group of infected animals in comparison with control group supplemented with alginite (NC2) with respect to weight of lungs and left kidney (*P* < 0.01), and right kidney (*P* < 0.001).

**Table 1:** Body weight (g) and the organ dimensions (g/kg) of the BALB/c mice on day 14 of application of additives

Group	The organ dimensions g/kg						Body weight (g)
	Heart	Liver	Spleen	Right kidney	Left kidney	Lungs	
NC1	6.51 ± 0.19	53.48 ± 0.92	4.92 ± 0.15	7.62 ± 0.25	7.65 ± 0.20	7.40 ± 0.19	18.64 ± 0.26
NC2	5.95 ± 0.14	55.60 ± 0.70	4.60 ± 0.09	7.29 ± 0.14	7.40 ± 0.15	7.90 ± 0.18	18.38 ± 0.27
IC	6.14 ± 0.20	75.00 ± 1.67 ***NC1,NC2	15.54 ± 1.40 ***NC1, NC2, *LAB	8.74 ± 0.21 **NC1,***NC2, *LAB	8.45 ± 0.22 *NC1,**NC2	10.37 ± 0.90 ***NC1, **NC2	16.87 ± 0.45 **NC1, *NC2
LAB	5.51 ± 0.18 **NC1	73.56 ± 1.99 ***NC1,NC2	11.94 ± 1.14 ***NC1,NC2	7.85 ± 0.21	7.84 ± 0.17	8.63 ± 0.22	17.35 ± 0.37

The results are expressed as the mean ± SD. Control NC1 (*n* = 16), control NC2 (alginite, *n* = 16), IC (alginite + *Salm.* Typhimurium CCM 7205_NAL_, *n* = 17), LAB (alginite + *Lact. reuteri* CCM 8617 + *Salm.* Typhimurium CCM 7205_NAL_, *n* = 17). *P < 0.05, **P < 0.01, ***P < 0.001

### Total Counts of *Lactobacillus reuteri* and *Salmonella* Typhimurium in Mice Faeces

**Fig. 1:**
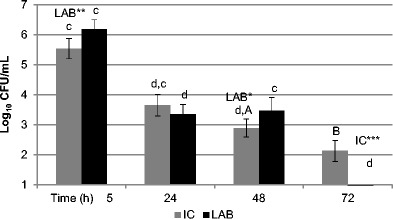
Plate count of *Salmonella* Typhimurium CCM 7205 in faeces of the BALB/c mice on day 7 post infection. Mice supplemented with alginite (IC, *n* = 17), the combination of alginite and *Lactobacillus reuteri* CCM 8617 (LAB, *n* = 17). The results are expressed as the mean log_10_ CFU/mL ± SD. *P < 0.05 (statistical differences between groups). ^A,B^*P* < 0.01; ^c,d^*P* < 0.001 (statistical differences within groups)

Throughout the experiment, the number of *Lactobacillus reuteri* applied to mice of the LAB group was maintained in the range of 8.11 ± 0.48–8.25 ± 0.21 log CFU/g of faeces. The total counts of *S.* Typhimurium in the faeces of experimental mice (Fig. 1) supplemented with both *Lactobacillus reuteri* CCM 8617 and alginite, and infected with *Salmonella* Typhimurium CCM 7205_NAL_ (LAB), determined at 5 h post infection, were significantly higher (*P* < 0.01) in comparison with group of infected mice supplemented only with fossile additive (IC). As soon as 24 h post infection, we detected a significant decrease (*P* < 0.001) in counts of the respective pathogen in samples of faeces of mice from both infected groups (IC, LAB) which declined to almost half of the counts determined at 5 h post infection. At this time, the total counts of *Salm.* Typhimurium in group LAB (3.35 ± 0.33 log_10_ CFU/mL) were lower by 0.30 log, however, they differed insignificantly from those of mice not supplemented with the probiotic strain (IC). While the total counts of *Salm.* Typhimurium in group LAB determined at 48 h post infection were the same as those at 24 h post infection (Fig. 1), the counts in group IC were significantly lower (by 0.76 log, *P* < 0.001) in comparison with those at 24 h post infection and, at the same time, significantly lower (*P* < 0.05) in comparison with group LAB. However, an opposite trend was observed at 72 h post infection (Fig. 1), when the most pronounced difference (*P* < 0.001) was observed between the two infected groups. The counts of *Salm.* Typhimurium in faeces of group LAB supplemented with *Lactobacillus reuteri* CCM 8617 and alginite were below the detection limit. At the same time, significantly lower counts of *Salm*. Typhimurium (*P* < 0.001) in comparison with those detected at 48 h post infection were observed. A decrease in pathogen counts at 72 h post infection was observed also in group IC, however, this decrease was not as pronounced as that in group LAB (*P* < 0.01). The counts in group IC at 72 h post infection were by 0.76 log lower in comparison with those at 48 h post infection.

### *Salmonella* Typhimurium Translocation

**Fig. 2:**
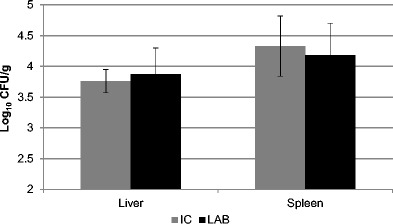
Translocation of *Salmonella* Typhimurium CCM 7205 in livers and spleens of the BALB/c mice on day 7 post infection. Mice supplemented with alginite (IC, *n* = 17), the combination of alginite and *Lactobacillus reuteri* CCM 8617 (LAB, *n* = 17). The results are expressed as the mean log_10_ CFU/g ± SD

After oral infection of mice with *Salm*. Typhimurium (groups IC and LAB), we observed a subsequent translocation of the pathogen from the digestion tract (Figs. 2) to parenchymatous organs (liver, spleen), and its highest although insignificantly different total counts (log_10_ CFU/g) varying around 10^4^ CFU were detected in the spleen of both infected groups of mice. The total counts of the investigated bacteria in the liver tissue in groups IC and LAB were lower by 0.31–0.57 log.

### Haematology Parameters

All parameters of the white component of blood determined in our study (Table 2) in mice from both infected groups (IC, LAB) significantly differed from those in control groups (NC1, NC2). The lowest absolute leukocyte (WBC) counts, well below the physiological limit [10], were observed in group IC (2.73 ± 0.47 G/L) and differed significantly (*P* < 0.01) from those in control mice not supplemented with additives (NC1), as well as from the control group (*P* < 0.001) supplied with fossile additive (NC2). Significantly lower (*P* < 0.001) and outside the physiological range were also the lymphocyte counts (Ly) and percentage of lymphocytes (Ly %), in comparison with both control groups (NC1, NC2). An opposite trend was observed for percentage of granulocytes (Gran %), which was the highest in group IC and significantly differed (*P* < 0.001) from all other investigated groups (NC1, NC2, LAB). Also the infected group LAB showed changes in white blood cell components, particularly in absolute WBC counts (*P* < 0.01) which were significantly lower in comparison with NC2, and in Ly counts (*P* < 0.01; *P* < 0.001) which were significantly lower in comparison with both control groups (NC1 and NC2, resp.). Similar as in group IC, Gran % was significantly (*P* < 0.001) increased also in group LAB in comparison with controls (NC1, NC2). Changes in white blood picture in the group supplemented with both the probiotic strain and fossile additive were, however, not as pronounced as in group IC and the results remained in the physiological range [10].

**Table 2:** Haematology parameters of the BALB/c mice on day 14 of application of additives

Group	NC1	NC2	IC	LAB	Ref BALB/c
WBC (G/L)	5.45 ± 0.42	6.19 ± 0.40	2.73 ± 0.47 **NC1, ***NC2	4.00 ± 0.41 **NC2	5.69–9.87
Ly (G/L)	4.04 ± 0.33	4.47 ± 0.29	1.29 ± 0.33 ***NC1, NC2	2.32 ± 0.29 **NC1, ***NC2	3.60–7.29
Mo (G/L)	0.17 ± 0.02	0.19 ± 0.02	0.11 ± 0.02	0.14 ± 0.02	0.34–0.70
Gran (G/L)	1.24 ± 0.13	1.53 ± 0.13	1.57 ± 0.25	1.54 ± 0.16	0.74–1.78
Ly %	73.89 ± 1.51	72.05 ± 1.13	42.01 ± 5.24 ***NC1, NC2	56.62 ± 2.10	55.06–73.44
Mo %	3.17 ± 0.32	3.38 ± 0.23	3.83 ± 0.14	4.24 ± 0.27 *NC1	3.75–7.26
Gran %	22.94 ± 1.23	24.58 ± 0.93	54.16 ± 5.16 ***NC1, NC2, LAB	39.14 ± 2.01 ***NC1, NC2,	10.46–18.94
RBC (T/L)	10.75 ± 0.52	9.73 ± 0.34	8.59 ± 0.75 *NC1	7.91 ± 0.43 ***NC1, *NC2	8.16–9.98
HGB (g/L)	185.8 ± 10.03	163.5 ± 5.61	140.2 ± 10.79 **NC1	131.3 ± 7.27 ***NC1, *NC2	124–154
HCT %	60.19 ± 3.03	54.11 ± 1.97	46.83 ± 4.15 *NC1	43.58 ± 2.36 ***NC1, *NC2	43.5–55.4
MCV (fL)	55.95 ± 0.33	55.61 ± 0.19	54.51 ± 0.21 **NC1, NC2	55.08 ± 0.21	50.8–55.6
MCH (pg)	17.17 ± 0.25	16.79 ± 0.20	16.51 ± 0.53	16.55 ± 0.19	13–15.5
MCHC (g/L)	307.3 ± 3.95	302.7 ± 3.68	304.0 ± 9.52	301.1 ± 3.32	239–280
PLT (G/L)	391.2 ± 88.43	512.0 ± 52.54	154.6 ± 39.88 **NC2	265.8 ± 50.09 *NC2	476–963

Control NC1 (*n* = 16), control NC2 (alginite, *n* = 16), IC (alginite + *Salm.* Typhimurium CCM 7205_NAL_, *n* = 17), LAB (alginite + *Lact. reuteri* CCM 8617 + *Salm.* Typhimurium CCM 7205_NAL_, *n* = 17), *WBC* white blood cells, *Ly* lymphocytes, *Mo* monocytes, *Gran* granulocytes, *RBC* red blood cells, *HGB* haemoglobin, *HCT* haematocrit, *MCV* mean corpuscular volume, *MCH* mean corpuscular haemoglobin, *MCHC* mean corpuscular haemoglobin concentration, *PLT* thrombocytes, *Ref* reference range [10]. The results are experience as the mean ± SD. *P < 0.05, **P < 0.01, ***P < 0.001

Although the red blood component in animals from both infected groups (Table 2) showed no pronounced deviations from the physiological range, significant differences in comparison with control groups were observed particularly in erythrocyte counts (RBC), concentration of haemoglobin (HGB), and the values of haematocrit (HCT). Experimental group LAB showed significantly lower proportion of RBC, lower concentration of HGB and HCT value in comparison with control groups NC1 and NC2 (*P* < 0.001; *P* < 0.05). Significantly lower counts of RBC and lower value of HCT (*P* < 0.05), and lower concentration of HGB (*P* < 0.01) was observed also in infected group IC in comparison with control not supplemented with additive (NC1). In both infected groups we observed also significantly lower counts of thrombocytes (IC: *P* < 0.01; LAB: *P* < 0.05) in comparison with group NC2.

### Biochemical Parameters

#### Enzymatic Profile

The most pronounced changes in the activity of hepatic enzymes and ALP were recorded on day 7 post infection (Table 3) in both infected groups (IC, LAB). While the mice from group IC showed an insignificant increase in non-specific liver enzyme AST and its activity exceeded the physiological limit of BALB/c mice (3.43 ± 0.61 μkat/l), the infected group supplemented with additives (LAB) exhibited an opposite trend. The activity of this enzyme in mice from group LAB was significantly lower (*P* < 0.05) in comparison to that in both control groups (NC1, NC2) and infected group IC (*P* < 0.01). The liver enzyme ALT is found only in the cytoplasm and its increase is associated with disturbances of hepatic cells membranes, even at the absence of necrosis. ALT is secreted in association with both reversible and irreversible damage to hepatic parenchyma. On day 7 post infection, we recorded an increased activity of ALT which exceeded the physiological limit, not only in infected but also in control mice. The increase in the activity was significantly higher (*P* < 0.05) in the infected group IC in comparison with control group NC2. Both infected groups (IC, LAB) showed most pronounced changes in the activity of ALP which was significantly lower (*P* < 0.001) in comparison with both control groups (NC1, NC2).

**Table 3:** Biochemical parameters of the BALB/c mice on day 14 of application of additives

Group	NC1	NC2	IC	LAB	Ref BALB/c
AST (μkat/L)	2.88 ± 0.38	2.87 ± 0.11	3.43 ± 0.61	0.88 ± 0.16 *NC1,NC2, **IC	2.62–3.05
ALT (μkat/L)	5.04 ± 0.39	4.14 ± 0.67	7.77 ± 1.15 *NC2	6.47 ± 0.48	0.68–2.89
ALP (μkat/L)	5.36 ± 0.24	4.90 ± 0.21	1.79 ± 0.25 ***NC1, NC2,	1.75 ± 0.29 ***NC1, NC2	1.83–6.23
Total protein (g/L)	71.02 ± 2.81	69.58 ± 2.70	57.12 ± 2.14 **NC1,*NC2, ***LAB	76.03 ± 3.33	60.8–73.0
Urea (mmol/L)	4.46 ± 0.36 **NC2	7.49 ± 0.92	4.15 ± 0.30 ***NC2, LAB	6.89 ± 0.15	5.70–7.14
Albumin (g/L)	35.28 ± 0.46	32.53 ± 2.86	25.30 ± 1.12 *LAB, ***NC1,**NC2	30.25 ± 0.92 *NC1	31.0–37.0
Triacylglyceride (mmol/L)	2.30 ± 0.06	2.75 ± 0.14	2.00 ± 0.05 **NC2	2.56 ± 0.19 *IC	up to 3.42
Cholesterol (mmol/L)	3.37 ± 0.04	3.44 ± 0.05	3.49 ± 0.11	2.97 ± 0.13 *NC1,NC2,**IC	2.09–3.65
HDL cholesterol (mmol/L)	1.57 ± 0.02	1.58 ± 0.02	1.54 ± 0.06	1.38 ± 0.03 *NC1,NC2	up to 1.78
LDL cholesterol (mmol/L)	0.81 ± 0.09	0.72 ± 0.03	1.13 ± 0.10 *NC2	0.87 ± 0.13	up to 0.38

Control NC1 (*n* = 16), control NC2 (alginite, *n* = 16), IC (alginite + *Salm.* Typhimurium CCM 7205_NAL_, *n* = 17), LAB (alginite + *Lact. reuteri* CCM 8617 + *Salm.* Typhimurium CCM 7205_NAL_, *n* = 17), *AST* aspartate aminotransferase, *ALT* alanine aminotransferase, *ALP* alkaline phosphatase, *Ref* reference range [10]. The results are expressed as the mean ± SD. *P < 0.05, **P < 0.01, ***P < 0.001

#### Nitrogen Profile

The most pronounced changes in nitrogen profile (Table 3) which involved total proteins (TP), albumin (ALB) and urea, were observed on day 7 post infection in the infected group supplemented with fossile additive (IC). Decreased exogenous uptake of feed by animals in this group was reflected in low levels of nitrogen profile which decreased below the physiological limit of mice of BALB/c line. In these mice we observed significantly lower levels of TP (*P* < 0.01; *P* < 0.05; *P* < 0.001) and ALB (*P* < 0.001; *P* < 0.01; *P* < 0.05) in comparison with other groups (NC1, NC2 and LAB, resp.). At the same time, a significantly lower level (*P* < 0.001) of urea was observed in this group in comparison with control group NC2 and infected group LAB.

#### Lipid Profile

On day 14 of the experiment (Table 3), we observed increased concentrations of triacylglycerides (TAG) and LDL-cholesterol which exceeded the physiological limit [10], and indicated increased exogenous intake of fat, most likely caused by ad libitum feeding of mice. The significantly lower level of TAG (*P* < 0.01) observed in infected group IC in comparison to control group NC2 only reflected the decreased uptake of feed after infection. Despite that, the level of LDL-cholesterol in this group was the highest and significantly (*P* < 0.05) differed from the level in control group NC2. The mice from infected group supplemented with additives (LAB) showed increased appetite in comparison with group IC and, as a result, also significantly higher (*P* < 0.01) level of TAG. Despite the higher intake of fat by mice in group LAB, this group exhibited significantly lower (*P* < 0.05) level of total cholesterol and HDL-cholesterol in comparison with both control groups (NC1, NC2), and lower level (*P* < 0.01) of total cholesterol in comparison with IC group.

### Concentrations of SCFAs in Caecum and Faeces

**Fig. 3:**
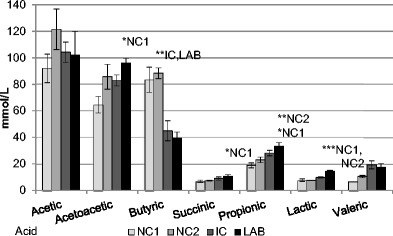
The caecum concentration of organic acids of the BALB/c mice on day 14 of application of additives. Control NC1 (*n* = 16), control NC2 (alginite, *n* = 16), IC (alginite + *Salm.* Typhimurium CCM 7205_NAL_, *n* = 17), LAB (alginite + *Lact. reuteri* CCM 8617 + *Salm.* Typhimurium CCM 7205_NAL_, *n* = 17). Results are expressed as mean ± SD. (*P < 0.05, statistical differences between groups)

Relatively high production of acetic acid was observed in the caecum of mice (Fig. 3) from all observed groups (NC1, NC2, IC, LAB). The insignificantly highest concentration of this acid was determined in control group NC2 which was higher by 19.3–29.44 mmol/L in comparison with other groups. This group of mice showed also relatively high level of butyric acid which differed significantly (*P* < 0.01) from that determined in both infected groups (IC, LAB). Investigation of proportions of individual organic acids in the caecum (Fig. 3) of mice from group supplemented with both *L. reuteri* and alginite (LAB) on day 7 post infection with *Salmonella* Typhimurium showed significant changes in the levels of propionic, lactic, acetoacetic and succinic acids. This group of mice also showed significantly higher (*P* < 0.05) production of acetic acid in comparison with control group NC1, which reached 95.95 ± 3.65 mmol/L. In the caecum of these animals, we detected also significantly higher production of lactic acid (*P* < 0.001) in comparison with control groups (NC1, NC2), and higher concentration of succinic acid (*P* < 0.05) in comparison with NC1, however, the levels of these acids did not exceed 15 mmol/L. The production of propionic acid in group LAB was significantly higher (*P* < 0.05; *P* < 0.01, resp.) in comparison with control groups NC2 and NC1, and reached 33.47 mmol/L.

The highest concentration of *lactic acid* (Table 4) in mice faeces was determined in group LAB on day 1 after the beginning of supplementation of mice with *Lact. reuteri* and alginite. It was significantly higher (*P* < 0.01) in comparison with groups NC1, NC2 and IC (*P* < 0.001). In the subsequent period, the levels of lactic acid in this group declined up to day 10 of our study with significant decrease observed on day 7 of study (*P* < 0.001). An increase in the level of lactic acid in faeces of mice from group LAB was recorded on day 7 post infection and the level of lactic acid on this day differed significantly (*P* < 0.05) in comparison with day 3 post infection. While the level of lactic acid in faeces of control mice (NC1) during days 1–14 of the study was relatively even and ranged from 25.29 to 28.33 mmol/L, the level of this acid in control group NC2 supplemented with fossile additive increased gradually and reached significantly higher concentration (*P* < 0.05) between days 1 to 10 of supplementation.

An insignificantly highest production of *acetic acid* (Table 4) during the 7 days pre-infection period, when the mice were supplemented with fossile additive, was observed in group IC. The level of acetic acid in the faeces of this group reached 11.45 mmol/L, which was higher in comparison with the level in group LAB. Similarly, a positive influence of fossile additive on production of acetic acid was observed in post infection period in group NC2 which exhibited a significantly higher level (*P* < 0.01) of acetic acid on day 3 post infection in comparison with infected groups (IC, LAB), and also in comparison with additive-free control group NC1 (*P* < 0.001). The increase in production of acetic acid in group NC2 depended on the duration of application of fossile additive as it significantly differed on days 10 and 14 (*P* < 0.001 and *P* < 0.01, resp.) in comparison with day 7 of supplementation. Production of acetic acid in mice supplemented with *Lact. reuteri* and alginite (LAB) before and after infection was uniform and the levels of this acid were relatively high (60.97–73.99 mmol/L).

Dynamics of concentration of *propionic acid* (Table 4) in the faeces of group NC2 was similar to that recorded for acetic and lactic acids and showed a gradual increase that reached significantly different level (*P* < 0.05) on day 7 of supplementation of alginite. A significantly highest production of propionic acid (*P* < 0.01) was detected in group LAB on day 1 of supplementation of additives to mice in comparison with group NC2. Subsequently, on day 7 of the study, group LAB exhibited similar decrease in propionic acid as that observed with lactic and acetic acids and the decrease was significant (*P* < 0.05). In the period post infection, mice from group LAB showed again increase in production of propionic acid that reached insignificantly different level on day 7 post infection (32.93 ± 3.65 mmol/L). While the production of propionic acid in group IC increased up to day 7 of supplementation of alginite, it showed an insignificant decrease in the post infection period, resembling that of lactic and acidic acids observed in this group.

The highest production of *succinic acid* (Table 4) in the pre-infection period was recorded on days 1 and 7 of the study in the group supplemented with *Lact. reuteri* and alginite (LAB) and in group IC supplemented with fossile additive. The increase in the level of succinic acid on day 1 after supplementation of additives in group LAB was significant (*P* < 0.001) in comparison with group NC2. A positive influence of alginite on production of succinic acid was observed also in mice from control group NC2 which showed a significant increase (*P* < 0.001) in this acid on day 7 of administration of alginite in comparison with day 1. On day 3 post infection, we recorded lower concentrations of succinic acid in both infected groups (IC, LAB), and the level of this acid in group NC2 was significantly higher (*P* < 0.05) in comparison with group LAB. However, on day 7 post infection, the level of succinic acid in group supplemented with probiotic strain and alginite (LAB) was significantly increased (*P* < 0.05) in comparison with day 3 post infection as well as in comparison with groups NC1 and IC (*P* < 0.01) and group NC2 (*P* < 0.001).

The level of *acetoacetic acid* in faeces of control mice from group NC1gradually decreased (Table 4) up to day 10 of the study, the decrease being significant (*P* < 0.01) between days 1 and 10. An opposite trend was observed in control mice supplemented with fossile additive (NC2) which showed a most pronounced significant increase in acetoacetic acid (*P* < 0.01) between days 1 and 10 of the study. The difference was significant on day 3 post infection in comparison with groups LAB and IC (*P* < 0.05; *P* < 0.01, resp.). The highest production of acetoacetic acid was observed in group LAB on the first day of our study when the level of this acid was significantly higher (*P* < 0.01) in comparison with groups NC2 and IC. The positive effect of combination of the probiotic strain and alginite on production of acetoacetic acid in comparison with group not supplied with probiotic strain (IC) was manifested by higher concentrations of this acid throughout our observation. A significantly higher level (*P* < 0.001) of acetoacetic acid exceeding that in mice from group IC by 25.24 mmol/L was observed on day 7 post infection.

Dynamics of *butyric acid* in faeces of mice from both control groups NC1 and NC2 (Table 4) resembled that observed with production of acetoacetic acid. We also recorded a declining trend in production of butyric acid in group NC1 throughout the observation, with its level ranging from 27.55 to 19.97 mmol/L. An opposite trend was observed in the control group supplied with fossile additive (NC2) where the level of butyric acid increased and the increase was significant (*P* < 0.05) between days 1 and 14 of the study. Significantly different concentrations of butyric acid in group NC2 in comparison with infected groups IC and LAB were recorded on days 3 and 7 post infection (*P* < 0.01 and *P* < 0.05, resp.). Significant differences in concentrations of butyric acid in the pre-infection period were observed between group supplemented with NC2 and group LAB on days 1 and 7 of supplementation (*P* < 0.05 and *P* < 0.01, resp.). In the post infection period (Table 4), we detected similar but insignificant decrease in production of butyric acid in group IC, resembling that observed with lactic, acetic and propionic acids.

The concentrations of *valeric acid* in faeces of mice from all groups were very low and did not exceed 20 mmol/l. Significantly different concentrations of this acid (*P* < 0.05) in comparison with infected group IC were observed in group NC2 after 10-day supplementation with alginite.

### Morphometric Parameters of Jejunum and Ileum

#### Jejunum

Comparison of cross sectional area of jejunal villi (Table 5) in mice from all investigated group showed that on day 7 post infection this area in group IC was insignificantly lower by 3310 μm^2^ (63,740 ± 2428 μm^2^) in comparison with that in control group NC1. At the same time, in the same intestinal section of group IC, we observed statistically insignificant lowest perimeter of villi (1260 ± 39.91 μm), lower by 22–75 μm in comparison with other groups. Similar trend in this group supplemented with fossile additive was observed also with the height of villi that was insignificantly lower by 12–29 μm in comparison with other groups (NC1, NC2, LAB). In the same intestinal section of mice from group IC, we observed a significantly higher depth of crypts (*P* < 0.05) in comparison with control group NC2, and a correspondingly lower villi/crypt ratio (3.98 ± 0.11). The continuous treatment of mice from group LAB with *Lactobacillus reuteri* CCM 8617 and alginite (Table 5) had a demonstrable positive influence on changes in the observed morphometric parameters. Except for the depth of crypts in group LAB (133.5 ± 1.38 μm), similar to that in group IC (132.6 ± 1.78 μm), there were differences in other parameters, particularly in cross sectional area and perimeter of villi in group LAB, which were bigger by 1510 μm^2^ and 22 μm, respectively, in comparison with infected group IC.

**Table 5:** Intestinal morphology of the BALB/c mice on day 14 of application of additives

Group	Cur surface of villi μm^2^	Villus perimeter μm	Villus height μm	Crypt depth μm	Ratio Villus height/Crypt depth
Jejunum					
NC1	67,050 ± 2518	1322 ± 42,59	559,3 ± 20,95	128,5 ± 1,18	4,35 ± 0,14
NC2	67,300 ± 2230	1335 ± 44,14	561,2 ± 18,56	127,3 ± 1,36	4,41 ± 0,17
IC	63,740 ± 2428	1260 ± 39,91	532,2 ± 20,02	133,5 ± 1,38 ^*NC2^	3,98 ± 0,11
LAB	65,250 ± 1393	1282 ± 23,31	544,2 ± 11,6	132,6 ± 1,78	4,11 ± 0,07
Ileum					
NC1	52,180 ± 2205	1077 ± 36,59	434,5 ± 18,38	137,7 ± 1,23	3,15 ± 0,11
NC2	53,030 ± 2394	1088 ± 40,83	441,0 ± 20,07	135,2 ± 1,54	3,26 ± 0,11
IC	49,200 ± 1762	1023 ± 28,41	409,0 ± 14,68	139,7 ± 1,33	2,93 ± 0,08
LAB	50,790 ± 1757	1042 ± 29,16	422,4 ± 14,67	139,4 ± 1,75	3,03 ± 0,07

Control NC1 (*n* = 16), control NC2 (alginite, n = 16), IC (alginite + *Salm.* Typhimurium CCM 7205_NAL_, *n* = 17), LAB (alginite + *Lact. reuteri* CCM 8617 + *Salm.* Typhimurium CCM 7205_NAL_, *n* = 17). The results are expressed as the mean ± SD. *P < 0.05

**Table 4:** The faecal concentration of organic acids of the BALB/c mice on days 1, 7, 10 (day 3 post infection) and 14 (day 7 post infection)

Acid (mmol/L)	Group	Day			
		1	7	10	14
Lactic	NC1	28.33 ± 1.82 **IC,***NC2	25.29 ± 1.86	26.51 ± 1.53	25.42 ± 1.91
	NC2	15.13 ± 1.08^a^	19.63 ± 1.37	22.47 ± 1.27^b^	24.56 ± 2.39
	IC	18.86 ± 1.67	28.28 ± 3.65	23.91 ± 0.69	22.71 ± 2.46
	LAB	41.41 ± 3.17^c^ **NC1, ***NC2, IC	19.36 ± 1.97^d^	13.06 ± 1.29^a^	29.15 ± 4.03^b^
Acetic	NC1	64.86 ± 9.10	57.53 ± 5.86	51.72 ± 4.24	65.6 ± 3.95
	NC2	45.32 ± 4.42	52.11 ± 2.72^c,A^	85.48 ± 3.70^d^ **IC, LAB,***NC1	72.23 ± 3.40^B^
	IC	64.24 ± 7.16	76.49 ± 18.06	56.48 ± 6.18	55.17 ± 8.29
	LAB	67.95 ± 7.26	65.04 ± 5.39	60.97 ± 4.14	73.99 ± 4.44
Propionic	NC1	33.91 ± 4.88	30.29 ± 1.82	24.50 ± 2.35	33.38 ± 3.49
	NC2	21.95 ± 2.06^a^	30.05 ± 1.87^b^	30.79 ± 2.03	29.08 ± 2.65
	IC	26.79 ± 3.60	29.35 ± 3.34	22.87 ± 1.70	22.95 ± 4.60
	LAB	37.52 ± 3.22^a^ **NC2	26.06 ± 3.30^b^	28.58 ± 1.31	32.93 ± 3.65
Succinic	NC1	15.97 ± 1.79	17.02 ± 3.15	14.19 ± 1.74	16.72 ± 2.78
	NC2	6.98 ± 1.00^c^	17.50 ± 1.49^d^	18.60 ± 1.41 *LAB	13.65 ± 1.34
	IC	16.99 ± 2.30	21.86 ± 3.53	11.54 ± 1.69	18.30 ± 1.97
	LAB	20.40 ± 1.66 ***NC2	22.50 ± 3.47	13.74 ± 1.17 ^a^	32.37 ± 3.58^b^ **NC1, IC,***NC2
Acetoacetic	NC1	69.92 ± 5.54^A^	84.93 ± 2.93	73.58 ± 3.52^B^	75.94 ± 4.17
	NC2	60.17 ± 4.16^A^	69.76 ± 2.58	79.26 ± 2.81^B^ *LAB, **IC	75.82 ± 2.72
	IC	58.16 ± 3.61	66.69 ± 7.34	61.55 ± 3.75	54.34 ± 3.64
	LAB	87.75 ± 4.88	70.82 ± 4.56	63.37 ± 3.68	79.58 ± 3.02 ***IC
Butyric	NC1	27.55 ± 2.49	24.26 ± 2.83	21.88 ± 2.07	19.97 ± 2.16
	NC2	17.70 ± 1.38^a^	23.92 ± 2.21	27.56 ± 2.14 **IC, LAB	29.30 ± 4.42^b^ *IC, LAB
	IC	25.61 ± 3.35 *LAB	27.56 ± 3.42 **LAB	15.60 ± 1.56	16.17 ± 3.16
	LAB	16.38 ± 0.52	15.77 ± 1.19	14.30 ± 1.37	17.43 ± 1.14
Valeric	NC1	14.74 ± 1.08	15.36 ± 2.70	11.01 ± 1.28 *IC	15.11 ± 1.11
	NC2	14.01 ± 1.02	12.36 ± 0.95	17.34 ± 2.53	16.23 ± 5.07
	IC	12.31 ± 1.32	9.86 ± 1.14	9.59 ± 1.00	7.42 ± 1.10
	LAB	12.52 ± 1.15	11.95 ± 1.16	11.96 ± 0.66	9.63 ± 0.55

Control NC1 (*n* = 16), control NC2 (alginite, *n* = 16), IC (alginite + *Salm.* Typhimurium CCM 7205_NAL_, *n* = 17), LAB (alginite + *Lact. reuteri* CCM 8617 + *Salm.* Typhimurium CCM 7205_NAL_, *n* = 17). The results are expressed as the mean ± SD. *P < 0.05, **P < 0.01, ***P < 0.001 (statistical differences between groups). ^a,b^*P* < 0.05, ^A,B^*P* < 0.01, ^c,d^*P* < 0.001 (statistical differences within groups)

#### Ileum

Similar trend as that found in the jejunum of investigated mice was observed also in the ileum of these animals. On day 7 post infection, we observed the lowest insignificant levels of all investigated parameters in the ileum of mice from group IC (Table 5) with the exception of the depth of crypts (139.7 ± 1.33 μm) that was almost identical with that in group LAB (139.4 ± 1.75 μm). Treatment with probiotic strain *Lact. reuteri* in combination with alginite alleviated the effect of *Salmonella* Typhimurium on morphometric parameters of the digestive tract in mice from group LAB. In comparison with infected group IC, mice from group LAB possessed villi which exhibited increase in cross-section by 1590 μm^2^, in perimeter by 19 μm and in the height of villi by 13 μm.

In both investigated sections of the digestive tract (jejunum, ileum) of control mice (Table 5) continuously supplemented with fossile additive (NC2), we observed positive differences in morphometric parameters in comparison with control group which received no additive (NC1). Supplementation of alginite for 14 days resulted in an increase in the cross sectional area of jejunal villi by 250 μm^2^, and ileal villi by 850 μm^2^ in group NC2 in comparison with control group NC1. We recorded also increase by 11–13 μm in the perimeter of villi in the jejunum and ileum of mice from the same group (NC2) in comparison with group NC1.

### Histological Evaluation of Liver Sections

**Fig. 4:**
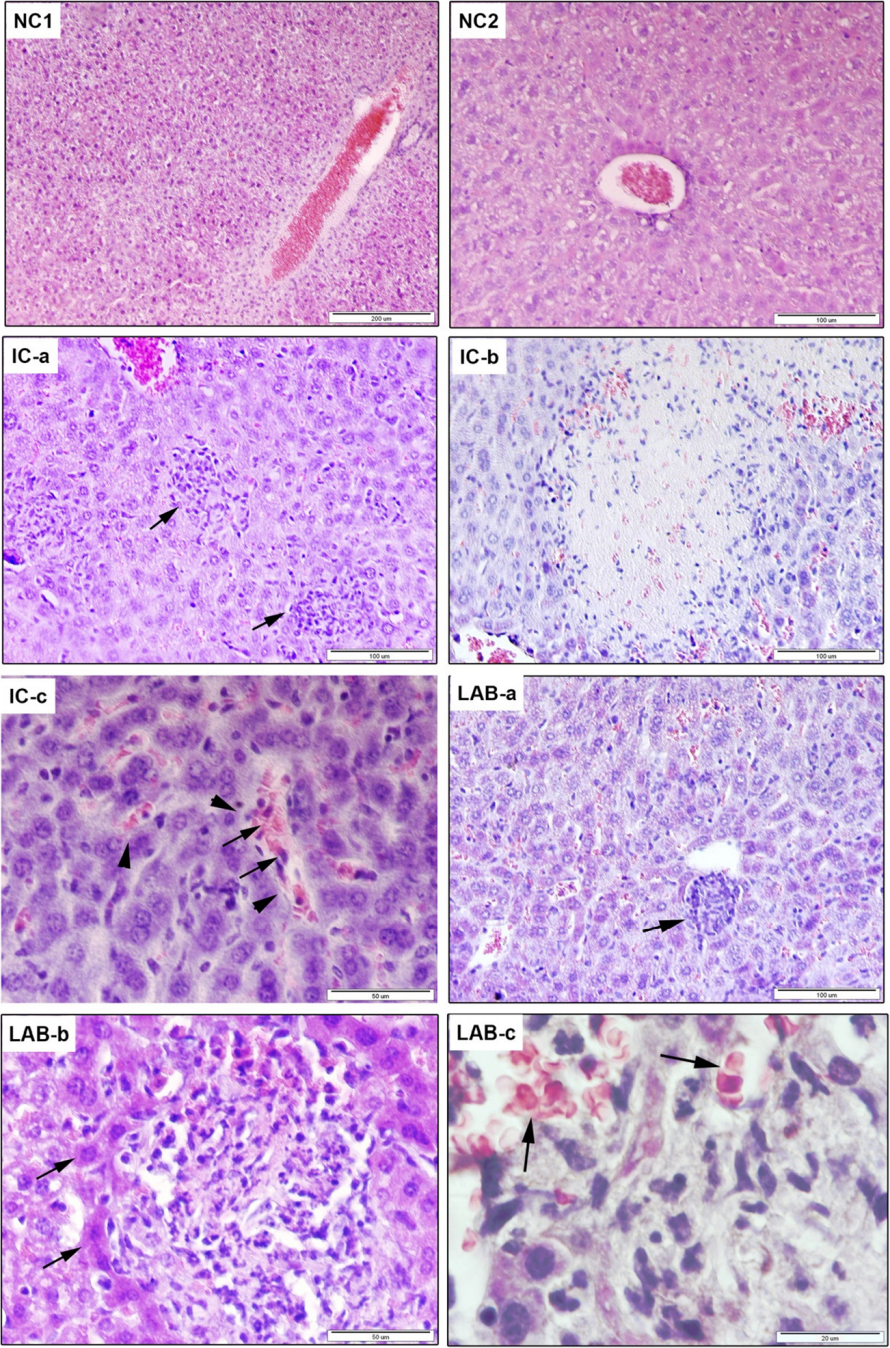
Representative histological images of haematoxylin and eosin stained sections of livers (NC1)-non-infected control group, (NC2)-non-infected control group 2, (IC a-c)-*Salmonella* Typhimurium CCM 7205 infected group, (LAB a-c)-infected group treated with alginite and *Lactobacillus reuteri* CCM 8607

Tissue architecture of normal livers of mice in non-infected control group (NC1) showed typical hepatic lobules consisting of hepatocytes with a prominent nucleus arranged around the central vein (Fig. 4, NC1). Feed supplementation of alginite to mice in non-infected control group NC2 did not cause any histopathological changes in the liver tissue morphology, which was normal in appearance (Fig. 4, NC2).

The liver sections of mice from group IC infected with *Salmonella* Typhimurium CCM 7205 showed prominent inflammatory changes and destruction of hepatocytes indicating infective injury to the liver (Fig. 4, IC a-c). The typical observations were clusters of lymphocytes and mononuclear cells referred to as “typhoid nodules”, that were localised next to the veins and in the liver parenchyma (IC-a). In the case of advanced degeneration of tissue due to cell necrosis/apoptosis, the pathologically advanced typhoid nodules (granulomas) contained amorphous tissue and abscesses (IC-b). The dilated sinusoids (arrowhead), sometimes filled with inflammatory cells (arrows), represented another pathological change and were seen inside or close to inflammatory nodules (IC-c).

The livers of infected mice which were treated with both alginite and *Lactobacillus reuteri* CCM 8607 (LAB) showed marked alleviation of overall inflammation and hepatocyte necrosis. This was demonstrated by reduced area occupied by typhoid nodules (arrow), probably as a result of reduced bacterial load (Fig. 4, LAB-a). Some inflammatory nodules were surrounded by normal hepatocytes with prominent nuclei and cytoplasm (arrows) and comprised various types of inflammatory cells, necrotic tissue and/or abscesses (LAB-b). Dilated sinusoids were still present in the livers in LAB group (arrows) and, except for macrophages and lymphocytes, also granulocytes were seen in the inflammatory lesions (LAB-c).

**Fig. 5:**
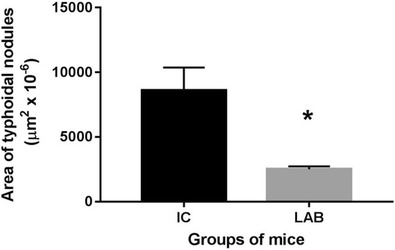
Morphometric analysis of the area occupied with typhoidal inflammatory nodules determined on the sections of livers of mice in IC and LAB groups (*n* = 4 for both groups). Measurements were performed on field screen, corresponding to the area of 0.146 μm^2^. Results are expressed as mean ± SEM. (*P < 0.05)

Liver sections of groups IC and LAB were morphometrically examined for the area occupied by typhoid inflammatory nodules, and the mean area ± SEM of livers from 4 mice/group are shown in Fig. 5. We recorded a significantly reduced mean area of nodules in the livers in LAB group (2505 ± 113 × 10^−6^ μm^2^) in comparison with IC group (8595 ± 887 × 10^−6^ μm^2^) (*P* < 0.05). The analysis was done on square areas (0.146μm^2^) of field screen.

## Discussion

The available literature refers to a wide spectrum of positive effects of humic substances on live organisms, ranging from their stimulating effect on digestion and utilisation of nutrients [4, 7, 11], improvement of production parameters such as intensity of growth, feed conversion and index of effectiveness of fattening [12, 13], up to their absorption and detoxication abilities and a role in prevention and therapy of many diseases [14–18]. For this study of modulation of intestinal bacteria found in GIT of model animals by a combination of beneficial bacteria and alginite, we selected SPF mice of BALB/c line. As far as the selection of pathogen was concerned, we focused on micro-organisms that act at the level of gastrointestinal tract of mice, significantly affect their health and by their presence and action raise productive and/or economic problems. Mice challenged with *Salmonella enterica* serovar Typhimurium represent a good model for the study of the protective and therapeutic effects of probiotic bacteria against intracellular enteropathogens [19]. Probiotics, such as *Saccharomyces boulardii, Saccharomyces cerevisiae, Escherichia coli* EMO, *Bifidobacterium longum*, *Bifidobacterium lactis*, *Bifidobacterium bifidum, Enterococcus faecium* and *Lactobacillus bulgaricus* confer a protective effect on the challenged mice [20–23].

In our study, the conventional SPF mice of BALB/c line were continuously supplemented with a probiotic (*Lactobacillus reuteri* CCM 8617) in a lyophilised form (0.05% in feed) in combination with alginite added to feed in 10% concentration. *Salmonella* Typhimurium CCM 7205_NAL_, resistant to nalidixin, was used as the infectious strain administered at a dose of 0.1 mL per os (10^8^ CFU/mL). Studies on the influence of various strains of probiotics which could prevent salmonellosis in a mouse model were conducted by a number of authors [24–26]. Frizzo et al. [24] observed inhibitory effects of a combination of probiotics (*Lactobacillus casei* DSPV 318T, *Lactobacillus salivarius* DSPV 315T and *Pediococcus acidilactici* DSPV 006T) in the study on mice of Swiss line using *Salmonella* serotype Dublin DSPV 595T as a pathogen. In another study, Andino et al. [25] investigated two strains of probiotics obtained from poultry faecal samples (*Lactobacillus acidophilius* and *Pediococcus* spp.) with respect to their potential to prevent salmonellosis in a mouse model. In this study 15 *Salmonella* strains were used including 3 strains of *Salmonella* Typhimurium (*Salm.* Typhimurium DT104; *Salm.* Typhimurium ATCC 14028; *Salm.* Typhimurium ATCC 23595) because this serovar causes disease in mice. The mice were infected with 0.25 mL of the bacterial suspension (10^8^ log CFU/ mL) by gastric gavage. Steinberg et al. [26] investigated safety and protective effectiveness of *Lactobacillus salivarius* (L38) and *Lactobacillus acidophilus* (L36) strains isolated from bovine stool samples in Swiss NIH mice using *Salmonella enterica* serovar Typhimurium as a pathogen. In our experiment, the animals were treated with approximately 7.87 × 10^5^ CFU bacteria *Lact. reuteri* CCM 8617 per mice, while in the study by Steinberg et al. [26] the mice were challenged by gavage with 0.1 mL of the bacterial suspension containing about 10^7^ CFU. In the study by Frizzo et al. [24], the mice were administered 10^9^ CFU of each probiotic bacterium (*Lact. casei* DSPV 318T, *Lact. salivarius* DSPV 315T and *Pediococcus acidilactici* DSPV 006T) and in the study by Andino et al. [25] the final concentration of probiotic bacteria was 10^9^ CFU, and these bacteria were provided daily in drinking water. There were differences also in the duration of administration of probiotics before infection with pathogen from the genus *Salmonella*. Frizzo et al. [24] and Steinberg et al. [26] used 10-day continuous administration prior to infection, Andino et al. [25] decided on 2-day and 10-day administration in 2 experiments and we administered the lactobacilli strain continuously for 7 days before infection.

After infection of mice, the same health changes were observed in these animals in our study as were in the study by Frizzo et al. [24]. The latter authors observed evident decrease in the intake of feed 48 h post infection in both infected groups of mice and a reduced body weight on days 4 to 7 post infection. A significant difference (*P* < 0.01) in body weight between both groups was detected on day 5 post infection with results in favour of the group treated with probiotics. Our experiments showed a reduced feed intake in the group continuously treated with *Lactobacillus reuteri* CCM 8617 and alginite 48 h post infection and in the group not supplemented with probiotic (IC) on day 6 post infection. The mice from infected groups (IC, LAB) exhibited lower total body weight and the decrease was significant in group IC in comparison with both control groups NC1 (*P* < 0.01) and NC2 (*P* < 0.05). The most pronounced difference in relative weights of both infected groups (IC, LAB) was observed in the weight of the liver and spleen which was significantly higher (*P* < 0.001) in these groups in comparison with both control groups of mice (NC1, NC2). Similar results were reported by Steinberg et al. [26] who observed a significant decrease in total body weight in infected groups of animals (*P* < 0.05) and, at the same time, 2 to 3-fold enlarged livers and spleens in comparison with control groups.

Similar to our study, Frizzo et al. [24] observed defined foci of coagulated necrosis with infiltration of polymorphonuclear leukocytes in histological sections of the liver of infected mice. However, our study showed a significantly lower (*P* < 0.05) intensity of inflammatory foci in the group supplemented with probiotics and alginite (LAB) and the foci were more condense contrary to group IC where we could observe in many cases undefined diffuse inflammation throughout the liver parenchyma. The positive influence of *Lactobacillus reuteri* CCM 8617 and alginite on liver parenchyma in comparison with IC group was confirmed by lower activity of the specific enzyme ALT and significantly reduced activity of non-specific hepatic enzyme AST (*P* < 0.01).

To exemplify, it was reported that *Lact. casei* DN-114-001 [27], *Lact.* sp. Dad13 [28] and *Lact. casei* CRL 431 [29] limited the translocation of *Salmonella* Typhimurium to spleen, liver and large intestine in a mice model and improved the health condition of animals. The favourable effect of probiotics on the liver parenchyma was observed also by Steinberg et al. [26]. They reported that salmonella-infected animals presented diffuse cell infiltrate that disrupted the normal lobular architecture of the liver and induced vacuolar degenerative changes, while both L38- and L36-treated and challenged animals better preserved the lobular architecture and hepatocytes aspects, despite the presence of parenchyma inflammatory cell infiltration in the liver.

The performance of probiotic bacterial strains differs because different bacteria have defined adherence sites, immunological effects, and varied effects in the healthy versus inflamed mucosal milieu [30]. Mice infected with *Salmonella* showed inflammatory changes in the caecum [31] and the small intestine [26]. These changes were manifested by the presence of submucosal oedema, oedematous changes in *lamina propria*, deepening of crypts, disruption of crypt architecture, reduced number of goblet cells, epithelial erosion or ulceration, infiltration of polymorphonuclear leukocytes into submucosa, *lamina propria*, epithelium and intestinal lumen. The results obtained in our study indicated a positive effect of continuous feed supplementation with probiotic bacteria *Lactobacillus reuteri* CCM 8617 and alginite in the periods before infection and for 7 days after infection on alleviation of infection induced by pathogenic bacteria *Salmonella* Typhimurium CCM 7205_NAL_.This was manifested not only by attenuation of lymphocytopenia, granulocytosis and thrombocytopenia, readjustment of nitrogen balance and reduced intensity of inflammatory foci in the liver parenchyma but also by positive influence on morphometric parameters of the small intestine.

With regard to the significantly positive properties of humic acids, such as their extremely low resorption in the gastrointestinal tract (up to 0.1%), their direct positive effect can be expected primarily on the intestinal level. Due to their macro-colloidal structure, humic acids provide good protection to peripheral capillaries and damaged mucosal cells [5, 6]. We assume that humic acids as a component of organic portion of alginite played an important role in protection of damaged mucosa, as such positive effect was detected in group LAB in our study in which we observed an insignificant increase in the cross-section of jejunal and ileal villi (by 1510 μm^2^ and 1590 μm^2^, resp.,) increase in perimeter of villi by 22 μm in the jejunum and 19 μm in the ileum, and increased height of villi in the ileum by 13 μm in comparison with the infected group IC. This hypothesis was also confirmed by our observations in the group of mice supplemented with alginite alone (NC2) which exhibited positive differences in morphometric parameters in both sections of the digestive tract (jejunum, ileum) in comparison with control mice which were not given any additives (NC1). Supplementation of alginite for 14 days resulted in increased cross-section of villi in NC2 group by 250 μm^2^ in the jejunum and by 850 μm^2^ in the ileum in comparison with group NC1.

Typically, *Salm.* Typhimurium in a mouse model will translocate across the intestinal tract and become systemic, infecting many organs. Furthermore, *Salmonella* persists for as long as 30 days post-inoculation, infecting organs but absent from the gastrointestinal tract [32]. In the study by Andino et al. [25], the results of 2-day administration of probiotics before infection with *salmonella* support these statements. However, it appears that in experiment 2, in which probiotics were administered for longer time (10 days) prior to infection, salmonella was capable of colonising the intestinal tract as the culturing recovered salmonella from faecal and intestinal samples [25]. The reason for this difference is unclear because the mice were given the same challenge dosage of salmonella in both experiments. Similar results supporting short-term colonisation of the digestion tract were observed also in our study, as significantly higher numbers of *Salm.* Typhimurium (*P* < 0.01) were found at 5 h post infection in the faeces of experimental animals treated for 7 days prior to infection with combination of *Lactobacillus reuteri* CCM 8617 and alginite (LAB) in comparison with the infected group treated only with fossile additive (IC). However, at 24 h post infection, we detected a significant decrease (*P* < 0.001) in faecal bacterial counts in both infected groups (IC, LAB), and these counts were almost by half lower in comparison with the counts at 5 h post infection. The most pronounced significant difference (*P* < 0.001) between both infected groups was observed at 72 h post infection with *Salmonella* Typhimurium CCM 7205_NAL_, when this pathogen could no more be detected in faeces of mice from group LAB.

Analysis of concentration and proportions of organic acids is important because of their proven bactericidal activity [33, 34]. Production of organic acids, particularly lactic and acetic, is affected by pH of the gastrointestinal tract, and favourably affects digestive processes. The study by Adams and Hall [35] provided proof of significant synergistic effect of lactic and acetic acids on inhibition of *Escherichia coli* and *Salmonella enteritis*. According to some authors, multiplication of *E.coli* [36, 37] and *salmonella* [38] is limited or even arrested at pH below 5. Momose et al. [39] observed that proportion of organic acids in the caecum of infected germ-free BALB/c mice is an important factor in relation to elimination of pathogenic *E. coli* O157:H7 from the digestion tract. In the study by Momose et al. [39], the counts of *Escherichia coli* O157:H7 in the caecum, colon and faeces were lower in the group of mice the microflora of which produced 12 mmol/L acetic acid and 12 mmol/L lactic at the level of caecum in comparison with the group where concentrations of acetic acid and propionic acid reached 12 mmol/L and 7 mmol/L, respectively, and the level of lactic acid was below the detection limit. Ogawa et al. [40] reported that 70 mmol/L of non-dissociated lactic acid sufficed to destroy representatives of *Escherichia coli* that produce Shiga toxin (STEC) strain 89020087, however, the 7.4 mmol/L level only suppressed their growth.

In our study we also used intestinal bacterium from the family *Enterobacteriaceae*, but from the genus *Salmonella.* With respect to proportion of individual organic acids in mice from group treated with *Lact. reuteri* and alginite (LAB), determined in the caecum on day 7 post infection with *Salmonella* Typhimurium, we detected relatively high concentration of acetic acid (102.2 mmol/L) and significantly different (*P* < 0.05) production of acetoacetic acid in comparison with control NC1. In the caecum of LAB mice we also observed a significantly higher production of lactic acid (*P* < 0.001) in comparison with control groups NC1 and NC2, as well as higher concentration of succinic acid (*P* < 0.05) in comparison with NC1, however, the levels of these acids did not exceed 15 mmol/L. The production of propionic acid in group LAB varied around the level of 33.47 mmol/L, and was significantly higher (*P* < 0.05; *P* < 0.01) in comparison with control groups NC2 and NC1, respectively. On day 7 post infection with *Salmonella* Typhimurium, we observed a positive effect on intestinal metabolism in group LAB in comparison with group IC, manifested by increased level of organic acids in mice faeces, particularly of acetic, lactic, propionic, succinic (P < 0.01) and acetoacetic (*P* < 0.01).

A higher nutritional burden in the caudal part of the small intestine stimulates a release of the polypeptidic hormone enteroglucagon from endocrine cells located in the mucosa [41, 42]. Some authors claimed that the production rate of crypt cells is related to plasma levels of enteroglucagon. The deepening of crypts may, however, partly be attributed to higher production of propionic, butyric and valeric acids in the colon that, by contrast, stimulates the production rate of crypt cells in the small intestine [43]. This opinion was confirmed also by our observations which showed optimum action of short-chain acids SCFA toward better absorption and digestion properties of the small intestine in group NC2. In the caecum of mice supplemented with fossile additive (NC2) we recorded significantly higher concentration of butyric acid (*P* < 0.01) in comparison to both infected groups (IC, LAB). The trend of increased production of SCFA observed in group NC2 depended on the duration of supplementation of fossile additive, as significantly different concentrations of acetic acid were observed on days 10 and 14 of supplementation (*P* < 0.001; *P* < 0.01) in comparison with day 7. We also observed a gradual increase in the level of lactic acid in this group, reaching significantly higher concentration (P < 0.05) between days 1 and 10 of treatment, as well as in the level of propionic acid (*P* < 0.05). On day 10 of additive supplementation, we recorded also higher levels of succinic (*P* < 0.05; *P* < 0.01) and butyric acids (*P* < 0.01) in comparison with groups LAB and IC.

While the probiotic strains utilised in the study by Andino et al. [25] did not afford the mice any protection, our study employing Multicolor FISH analysis of caecal samples [44] showed a significant increase in the counts of representatives of genera *Lactobacillus* and *Bifidobacterium* and a significant decrease in representatives of genera *Clostridium, Bacteroides* and *Enterobacteriaceae.* At the same time, we were able to observe a positive effect on the bacteria from the genus *Lact. reuteri.* Results of study by Slížová [44] showed that the treatment with selected strain in combination with alginite resulted in colonisation of jejunum, ileum and caecum and modulation of the investigated intestinal microflora. A study oriented on the influence on the structure of microflora in mice was presented by Fuentes et al. [45]. The authors observed that administration of *Lact. casei* and *Lact. plantarum* resulted in changes in proportion of lactobacilli (*Lact. helveticus, Lact. johnsonii* and *Lact. reuteri* dominated) in faeces and in individual intestinal sections, however, the structure and numbers of other microflora components were not affected which implies different effectiveness of individual strains or species of lactobacilli.

### Conclusions

The results obtained in our study indicated a positive effect of continuous feed supplementation with probiotic bacteria *Lactobacillus reuteri* CCM 8617 and alginite in the periods before infection and for 7 days after infection on alleviation of infection induced by pathogenic bacteria *Salmonella* Typhimurium CCM 7205.
